# Unprofessional or Admirable? Determinants of Purchasing Behavior in Government Officials’ Livestreamed Shopping

**DOI:** 10.3390/ijerph192013073

**Published:** 2022-10-11

**Authors:** Wenshan Guo, Ninghua Sun

**Affiliations:** College of Public Administration, Huazhong University of Science and Technology, Wuhan 430074, China

**Keywords:** livestreaming, stimulus–organism–response, government officials, purchase intention, impulsiveness

## Abstract

As a new form of poverty governance, government officials’ livestreaming e-commerce of agricultural and sideline products has been booming since the outbreak of the coronavirus disease (COVID-19) in 2019. However, exploring the determinants of consumer purchase intentions in the context of government officials’ livestreaming is still limited. Drawing on the stimulus–organism–response (S–O–R) framework, this study develops a research model to examine the effect of platform factor (information quality), user factor (bullet screen mutuality), and streamer factors (streamer trustworthiness, streamer expertise, and streamer responsiveness) on perceived information usefulness and arousal, which in turn affect purchase intention. This study also integrates impulsiveness as a moderator. We use structural equation modeling to analyze 430 samples. Our results show that perceived information usefulness and arousal have a significant positive influence on purchase intention. Moreover, impulsiveness moderates the relationship between perceived information usefulness and purchase intention and between arousal and purchase intention. Livestreaming features and streamer characteristics can activate these two mechanisms. This study provides theoretical contributions to livestreaming and the S–O–R literature, as well as practical insights into livestreaming government officials.

## 1. Introduction

The obstruction of traditional channels has led to a large number of unsold agricultural and sideline products since the outbreak of COVID-19 in 2019. To help farmers avoid poverty, government officials at all levels promote agricultural and sideline products using a livestreaming e-commerce method. Livestreaming e-commerce breaks the bottleneck of insufficient information interaction in traditional e-commerce and reduces the information asymmetry of transactions, thus improving consumers’ purchasing intentions [1]. As an e-commerce company, Alibaba data show that, in the first quarter of 2020, more than 600 mayors sold agricultural products through livestreaming to achieve sales of 6 billion CNY [2]. The data from TikTok show that government officials sold more than 2.475 million agricultural products through livestreaming in the first half of 2020, with sales exceeding 118 million CNY [3]. In 2020, “Thank you for making orders for Hubei” launched by CCTV media was only a 2 h livestreaming e-commerce on 6 April. The cumulative number of viewers reached more than 120 million, with total sales of about 40.14 million CNY [4]. The livestreaming e-commerce of agricultural and sideline products realizes the efficient docking of supply and demand through an innovative transaction model. The livestreaming e-commerce of government officials has helped farmers avoid poverty, improved the government image and social governance, and increased citizens’ trust in the government [5].

Compared to traditional e-commerce platforms for agricultural and sideline products, livestreaming e-commerce has innovative features. First, streamers actively attract purposeless consumers by dynamically displaying rich, diverse, interesting, and useful content and information, thereby creating consumer demand [6,7]. Second, livestreaming creates a “face-to-face” information interaction context that can more accurately and quickly respond to consumer demands and narrow the emotional distance with consumers [1]. Third, credible government officials, authoritative media, and celebrities livestreaming agricultural products have the advantage of quality endorsement. When consumers believe that the quality of agricultural products is safe and reliable, they are more inclined to make purchasing decisions. Fourth, as an innovative function of livestreaming, a bullet screen is the main way for viewers to interact with each other. Viewers can use bullet screen comments sent by others as shopping references [8]. Consumers can better evaluate products and make purchasing decisions by obtaining real-time, dynamic, and multidimensional information. In sum, livestreaming e-commerce enhances consumers’ shopping experience and materializes potential purchases by providing comprehensive information.

Livestreaming e-commerce has greatly exceeded e-commerce platforms and offline platforms, leading to the explosive growth of livestreaming platforms, such as Facebook and YouTube in the United States, and TikTok, Taobao, and Pinduoduo in China. Livestreaming e-commerce, as a new form of shopping, is becoming increasingly popular. However, thus far, livestreaming e-commerce research is still in its infancy. Existing research mainly examines the antecedents of consumers’ intention and motivation to purchase general products [1,6,9], the interaction between consumers and streamers (e.g., virtual gifts) [10], and consumer participation behaviors [11,12,13]. Unlike celebrities’ livestreaming e-commerce, the essence of government officials’ livestreaming e-commerce is to develop the rural economy and increase farmers’ income. To achieve the goal of rural revitalization, government officials have promoted rural specialty products through livestreaming [5]. Government officials’ livestreaming e-commerce has realized an efficient connection between farmers’ supply and citizens’ needs and has become an important method of poverty alleviation under an epidemic. However, studies on the role of government officials’ livestreaming e-commerce in influencing consumers’ purchasing intentions are lacking.

Previous research has taken gender [14], knowledge [15], and the need for belonging [16] as the moderators of consumers’ purchase intention. With an increase in shopping channels, Floh and Madlberger [17] found that impulsiveness, as an individual’s psychological characteristics, plays an important role in consumers’ decision-making behavior. Chang et al. [18] used the elaboration likelihood model (ELM) to explore the antecedents of consumers’ purchase intentions in the Facebook’s secondhand marketplace. They found that impulsiveness positively affects consumers’ purchase intentions. Conversely, Chopdar and Balakrishnan [19] found that impulsiveness negatively affects consumers’ purchase intentions in the context of mobile shopping. This contradictory result motivates us to investigate the role of impulsiveness in the context of livestreaming e-commerce. Furthermore, empirical research on the effect of impulsiveness on purchase intention in the context of livestreaming is just beginning and, therefore, limited.

Impulsiveness refers to an individual tendency that includes urges to engage in an unplanned action or to take action immediately without evaluation or deliberation of consequences [20]. The characteristics of impulsiveness differ among consumers. Individuals with higher discrepancies between their real self and their ideal self tend to purchase impulsively [21]. Existing studies have confirmed that, when highly impulsive consumers perceive that websites provide high-quality information, they are more likely to purchase products [18,19]. The streamer offers product information in real-time video during livestreaming. At the same time, consumers can ask for product information through a bullet screen, and the streamer can rely on livestreaming to provide consumers with personalized information and services. Therefore, when highly impulsive consumers watch livestreaming, they are more likely to purchase products without thinking because of the positive emotions generated by the livestreaming content, streamer, or livestreaming environment. However, research on the role of impulsiveness in the context of livestreaming is scarce.

Considering the abovementioned gaps, this study mainly addresses two questions: (1) What are the key antecedents affecting consumers’ purchasing intentions in the context of government officials’ livestreaming e-commerce? (2) Does impulsiveness affect consumers’ purchase intentions in the context of government officials’ livestreaming e-commerce?

To answer these research questions, this study uses the S–O–R framework to examine the effect of platform factor (information quality), user factor (bullet screen mutuality), and streamer factors (streamer reliability, streamer responsiveness, and streamer trustworthiness) on perceived information usefulness and arousal, which affect purchase intention. The moderating effect of impulsiveness is also examined. This study contributes to the existing literature. First, this study focuses on the predictors of purchasing agricultural and sideline products. Previous livestreaming e-commerce literature did not consider specific product categories. Second, credible government officials pay more attention to product quality. If the products purchased by consumers are expired, deteriorated, or crushed, the government’s image will be severely damaged, thus reducing citizens’ trust in the government. Third, the extant literature has integrated impulsiveness into various contexts for research. This is one of the few studies using the S–O–R paradigm to investigate the antecedents affecting consumer purchase intentions in the setting of livestreaming e-commerce.

The remainder of this paper is structured as follows: First, we search the literature on livestreaming e-commerce, impulsiveness, and the S–O–R framework in detail to naturally and smoothly elicit research questions. We then develop the research model and discuss the research methodology and empirical results. We present the theoretical and practical implications, limitations, and future research trends. Finally, the conclusions are presented.

## 2. Literature Review

To investigate whether and how government officials livestreaming e-commerce affects consumers’ purchase intention, we divide the literature review into three research streams that form the theoretical basis of this study. Firstly, we review the current state of research on livestreaming e-commerce. By comparing the characteristics of livestreaming e-commerce and traditional e-commerce, it is highlighted that livestreaming e-commerce is shown to have great potential value as an innovative service channel. In addition, we demonstrate the process of government officials’ livestreaming e-commerce to position our research. Secondly, we review the relevant literature on impulsiveness to enrich the research model. Lastly, we elaborate on the S–O–R framework used in the study and explain in detail how the theory can be applied to this study.

### 2.1. Livestreaming E-Commerce

As a new form of shopping, livestreaming e-commerce has real-time media and social commerce attributes. It has become one of consumers’ favorite forms of shopping, as it provides a real-time, authentic, and interactive environment [22]. In December 2020, China’s livestreaming commerce users numbered 388 million, which accounted for 39.2% of the total netizens, and an increase of 123 million over March 2020. Users who have purchased things through livestreaming e-commerce accounted for 66.2% of the total livestreaming e-commerce customers, of which 17.8% of the users’ livestreaming e-commerce consumption accounted for more than 30% of their online shopping consumption [23]. The fusion of livestreaming and marketing activities has prompted the emergence of two livestreaming business models: livestreaming embedded in e-commerce (Taobao live and JD live broadcast) and e-commerce integrated into livestreaming (TikTok and Kuaishou). Innovative business models provide consumers with a different shopping experience compared to traditional e-commerce platforms. As livestreaming e-commerce has the advantages of sharing content, meeting needs, enhancing experience, and stimulating consumer desire, various products, such as clothes, cosmetics, digital products, and books, have entered the livestreaming platform.

Livestreaming e-commerce provides consumers with comprehensive, timely, and accurate product information due to the characteristics of real-time interactions among multiple participants. It has significant advantages over other online shopping platforms. First, livestreaming e-commerce displays products in the form of videos. The video display form is more vivid and authentic than text and pictures, which may affect the audience’s cognition of the product [1,12]. Second, it enables users to communicate with the streamer in a “face-to-face” manner. Information source characteristics, such as the appearance, personality, and professionalism of the streamer may have a potential impact on the audience’s emotions, cognition, and behavior [24,25,26]. In addition, the streamer can share the product experience or feelings. Consumers can also consult and evaluate products on the bullet screen [8,27]. Third, viewers can judge other viewers’ attitudes toward the product and the streamer through real-time comments, likes, gifts, and purchases made by other participants, and then use these judgments to guide their own behavioral decisions [28]. Table 1 summarizes the differences between livestreaming e-commerce and e-commerce.

Livestreaming is a combination of real-time video content and text-based chat tools (bullet screen) [25]. Users use livestreaming for a wide range of purposes, such as knowledge sharing [29], games [30,31,32], and online sales [1,9]. Currently, the research on livestreaming e-commerce is still in its infancy. The existing literature focuses on the following two aspects of livestreaming e-commerce. The first aspect is the motivation of users to watch or participate in livestreaming e-commerce. Kang et al. [11] analyzed 3,500,445 online review texts by developing a text mining method. The results show that the interactivity of livestreaming has a curvilinear relationship with customer engagement behavior. Zhao et al. [33] used self-determination theory to explore the determinants of users’ continued use of Twitch livestreaming. The second aspect is the effect of livestreaming e-commerce on consumer behavior, such as purchase intention. Existing research uses different theories to explore the factors that affecting consumers’ purchase intentions [1,6,25], such as signaling theory [6], ELM [1], uses and gratification theory (U&G) [9,13], affordance theory [25], and network externality theory [9]. Although the streamer plays a vital role in livestreaming e-commerce, the existing literature does not examine the role of government officials as the streamer in the context of livestreaming e-commerce. In addition, most existing studies focus on the purchase intention of general products without considering the category of specific products.

In terms of methodology, most livestreaming studies adopt questionnaire survey, online experiment, text mining, data mining, and web crawling methods. Regarding the questionnaire method, Gao et al. [1] investigated the impact of central route factors and peripheral route factors of livestreaming commerce on consumers’ purchase intentions by distributing questionnaires on the Wenjuanxing platform. Drawing on signal theory and uncertainty literature, Lu and Chen [6] explored the impact of physical characteristics similarity and value similarity on consumers’ purchase intention in the context of clothing and cosmetics. Similarly, the questionnaire was distributed on Wenjuanxing website. In addition, the study used the interview method to investigate product-related and livestreaming-related factors that affect consumers’ purchase intentions. In terms of online experimental method, Tong et al. [13] developed a 3 (high vs. medium vs. low background visual complexity) × 2 (male vs. female) between-subject online experiment. By manipulating the number of colors, as well as the type and number of elements, the background visual complexity was divided into three dimensions: high, medium, and low. Participants were randomly assigned to one of the six scenes after watching the video. The results showed that the background visual complexity of the live broadcast room had a positive impact on consumers’ purchase intentions through emotional states (pleasure and arousal). Kang et al. [10] developed a text mining method. By analyzing 3,500,445 online review texts on Sina Weibo, they found that interactivity had an inverted U-shaped effect on customer engagement behavior. Tie strength plays a completely intermediary role between interaction and engagement behavior. Zhou et al. [26] explored the impact of danmaku-related stimulus types on paid gifts by crawling the data of DOUYU.COM. The results showed that presence of others, social competitions, emotional stimuli, and number of excitement-related words positively affect paid gifting.

**Figure 1:**
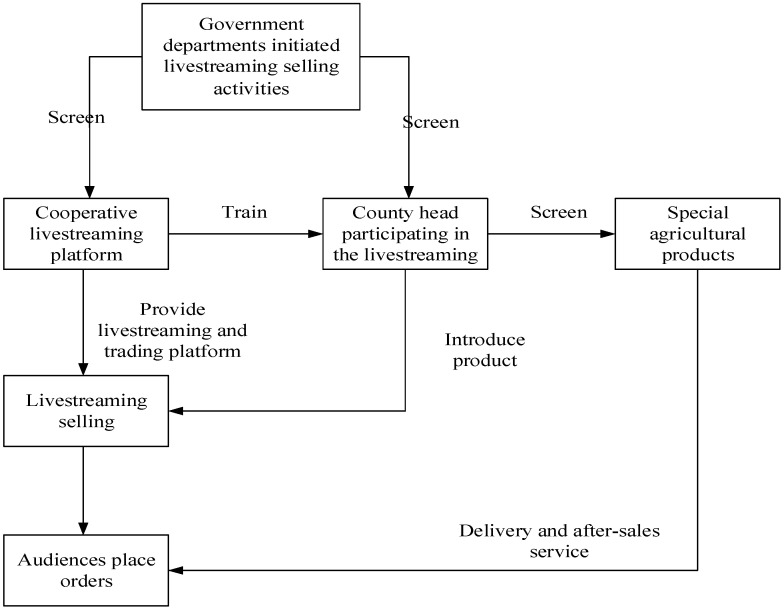
Flowchart of livestreaming of government officials.

To solve the problem of unsalable agricultural and sideline products, government officials have become streamers to promote products with the support of national policies. Unlike celebrities, government officials who act as streamers endorse the credibility of the government. When streamers deliver safe and reliable information to consumers, consumers increase their trust in the quality of agricultural products and have a greater tendency to make purchase decisions. The flow of government officials’ livestreaming e-commerce is shown in Figure 1. The specific process is divided into five steps. First, in compliance with national policies, government departments initiate all livestreaming e-commerce activities. The government staff screens poor counties and coordinates the livestreaming e-commerce platform. Second, the platform staff selects the products to be sold and makes publicity videos through the investigation and selection of agricultural and sideline products. The video is then widely publicized on livestreaming and social platforms, such as TikTok, Kuaishou, and Weibo. Third, the trainers provide live broadcasting skills training to the county heads participating in live broadcasting to ensure that the county heads promote the local characteristics of the agricultural products in a good state. Fourth, after the livestreaming selling event is officially launched, the county heads enter the livestreaming room to conduct the livestream by introducing the quality, features, functions, and methods of use of the products to the audience. The county heads also actively answer questions from viewers and encourage them to place orders. Fifth, after the viewers places the order, the relevant companies pack, ship, and provide an after-sales service to complete the entire transaction process.

### 2.2. Impulsiveness

Consumers’ personality traits are considered an important factor in explaining consumers’ impulse purchases [17,34,35]. Youn and Faber [36] considered that consumers’ impulse buying could be affected by environmental factors, and may also be affected by the internal state (i.e., emotion or arousal) or the individual characteristics experienced by consumers. Individual characteristics include impulsiveness, values, hedonism, pressure, and concentration [37]. Impulsiveness is the most commonly used personality trait to explain consumers’ impulsive purchase behaviors [38]. Environmental characteristics involve external cues of the shopping environment, such as the atmosphere [17,39]. Previous studies have shown that consumers’ impulsive traits have individual differences, which leads to different types of impulsive buying behaviors [40,41,42,43]. Stern [44] grouped impulse purchases into four categories: pure impulse buying, reminder impulse buying, suggestion impulse buying, and planned impulse buying. Consumers with high self-control are usually cautious, careful, rational, and planned. Conversely, highly impulsive consumers are often instinctive, reckless, spontaneous, nonreflective, and immediate [40]. They are more sensitive to the external environment and are more likely to buy impulsively stimulated by the external environment. As a personal trait, impulsiveness has been widely studied in the literature on e-commerce [43] and mobile commerce [19]. Personal traits are stable. In different shopping environments, individuals show consistent personality characteristics and behaviors. For example, e-commerce websites that provide high-quality information increase consumer impulse purchases [43,45]. In the context of mobile commerce, impulsiveness has a significant positive relationship with consumers’ buying and use behaviors [19]. In this study, livestreaming e-commerce with real-time video interaction enables the streamer to fully display the product and stimulates the audience’s love for and recognition of the streamer. When the audience faces the same external environment, i.e., all participants enter the livestreaming, viewers with different impulsive traits show different buying behaviors. Individuals who are highly impulsive are more likely to be aroused by stimulation, which affects their behavioral decisions. In other words, highly impulsive consumers may buy a product because of the product appearance or livestreaming environment, rather than their need for it. Therefore, it is necessary and timely to explore the role of impulsiveness in the research of consumers’ purchase behavior in the livestreaming e-commerce environment.

### 2.3. The Stimulus–Organism–Response Framework

Mehrabian and Russell [46] proposed a stimulus–organism–response (S–O–R) model in the context of environmental psychology. The S–O–R model believes that external stimulus cues can trigger individual emotions and cognitive processes, thereby acting on the user’s behavioral response [45,47]. This model contains three critical elements: stimulus, organism, and response. Stimulus refers to the external environmental factors that lead to the change of an individual’s internal state. Stimulus cues include various external cues, such as the national economic environment, the working environment of enterprises, and the consumption environment. Organism refers to the response of individuals stimulated by environmental factors. Organism response reflects a series of psychological activities generated by individuals under the stimulation of a specific external environment, including positive or negative emotions, such as excitement, immersion, disgust, and arousal [48,49]. Behavioral response refers to the user’s behaviors, such as use, participation, feedback, and purchase after environmental stimuli and psychological changes.

As a mature and stable model, S–O–R has been applied to various research backgrounds, such as mobile commerce [19,49], social commerce [50], gamification [51], advertising [52], and tourism [53]. The previous literature demonstrates that stimuli can be expressed in various forms, such as system features, content features, and consumer characteristics [54,55]. Organisms often use cognitive/affective reactions and positive/negative emotions to capture the internal state of individuals [51,56,57]. The response includes individual use, participation, feedback, purchase, and comment behavior [8,54,58]. Specifically, Baker, Grewal, and Parasuraman [59] found that environmental cues (i.e., appearance, decoration, and music) in retail stores can affect consumers’ judgments on product quality. Chopdar and Balakrishnan [19] proposed that technical characteristics of mobile shopping applications (ubiquity, visual attractiveness, incentives, and contextual offering) can stimulate users’ cognitive and affective reactions (perceived value and impulsiveness), which consequently affect their behavior (repurchase intention). Hsiao and Tang [53] employed the S–O–R model to explore the effects of media cues (content timeliness and media richness) and social cues (critical mass and social interaction) on players’ response (visiting intention and continue intention) through players’ organism (attachment and conformity) in the context of gaming and tourism.

Although the S–O–R model has been applied to multiple fields and has been studied many times, its application in the context of live broadcasting is limited. As livestreaming e-commerce and mobile commerce have different functions in many aspects, we use the S–O–R framework as a theoretical basis to examine the effect of platform, user, and streamer dimensions on consumers’ purchase intention. Two reasons can justify the application of S–O–R in this study. First, previous researchers applied the S–O–R framework to predict consumer purchase intention in the retail environment, and their findings support its applicability [19,49,50]. Second, it can parsimoniously and theoretically justify the examination of livestreaming dimensions as stimuli and is capable of evaluating the role that the consumer perception of livestreaming dimensions plays in consumer purchase intention.

In this study, environmental stimuli are composed of platform dimension (information quality), user dimension (bullet screen mutuality), and streamer dimension (streamer reliability, streamer responsiveness, and streamer trustworthiness). Information quality reflects the accuracy and reliability of information. In this study, consumers watching livestreams can receive product-related information from streamers and bullet screens. Complete, accurate, and timely information helps them better evaluate products. Bullet screen mutuality reflects the extent to which viewers in a live broadcast room are able to receive help from co-viewers in terms of knowledge and experience. During the live broadcast of the anchor, the audience can understand the anchor and product information through the bullet screen sent by the co-viewers. At the same time, viewers can send gifts to express their love for the anchor. The anchor is the main source of product information for the audience and is one of the main characteristics that distinguish livestreaming e-commerce from other forms of e-commerce. Government officials as anchors are endorsed by the credibility of the government, and their credibility is much higher than that of star anchors. Trained government officials also have the expertise and responsiveness to sell products. The existing literature suggests that these characteristics significantly affect consumers’ cognitive and affective states. Organisms reflect the internal experiences of consumers in the shopping process. After exposure to stimuli, consumers process them into helpful information and express emotions, such as pleasure and excitement. Therefore, this study considers perceived information usefulness and arousal to be organisms. Consistent with Cheng et al. [50], we use purchase intention as the final response of consumers.

## 3. Research Model and Hypotheses

Information quality refers to the measurement of how highly the quality of information provided by the system matches the needs of users [60]. Information quality encompasses system characteristics such as usefulness, comprehension, relevance, accuracy, and timeliness. High-quality information is easy to understand, accurate, timely, reliable, relevant, and complete [60,61,62]. Due to the information asymmetry between producers and consumers, high-quality product information plays an important role in consumers’ deliberated evaluation of the actual state of products [63,64]. Previous research has shown that high-quality information is useful and valuable. For example, when a social commerce website provides consumers with high-quality information about related products or services that they need in a transaction, consumers perceive the information and the website to be useful [60,63]. In addition, mobile commerce, which provides high-quality information, enables users to obtain the information they need promptly during the shopping process. Consumers who obtain high-quality information greatly reduce the time they spend on mobile shopping, thereby enhancing their perceived information usefulness [65,66]. Applying this logic, the information quality of this study refers to the extent to which government officials’ livestreaming provides consumers with complete, accurate and timely product information. Government officials, as streamers, show and provide product information in the form of videos, and they answer questions raised by consumers on the bullet screen in real time. When government officials provide consumers with complete (i.e., product details, experience sharing, and promotion information), accurate (i.e., clearly displays the actual state of the product), and timely (i.e., timely interaction with the audience and providing them with the latest product information) product information during the livestreaming, consumers believe that livestreaming content has high information quality. Consumers use the high-quality information obtained from livestreaming to evaluate products and guide purchase decisions. Therefore, high-quality livestreaming information makes consumers perceive the information as useful, helping them make informed decisions. Therefore, we hypothesize the following:

**Hypothesis** **1** **(H1).** *Information quality positively affects consumers’ perceived information usefulness.*

Mutuality refers to consumers’ exchange of shopping advice about a brand or the sharing of product information or recommendations with other consumers on a social media platform [67,68]. In view of the fact that mutuality can help peers solve product use problems by using social media platforms (i.e., Facebook, Instagram, and WeChat) to share product purchase experiences and suggestions, marketers, and managers invest significant resources to facilitate mutuality [69]. A bullet screen is a type of comment that scrolls on the screen in real time. It is the main way of interaction between viewers during livestreaming [1]. Compared to a traditional video bullet screen, the content of a livestreaming bullet screen has higher value to the audience. On one hand, the audience can watch the bullet screen information sent by the co-viewer in real time or obtain product information through the interaction between the co-viewer and the streamer [22]. On the other hand, the audience can also raise questions about product information (product size and price) through the bullet screen. By increasing their knowledge of the product, the audience can reduce product uncertainty due to information asymmetry [6]. In addition, mutuality can increase the positive arousal of users. When users obtain useful information from the barrage of information provided by their co-viewers, their favorability of the live broadcast increases, stimulating the audience’s psychological arousal. Pop-up messages (i.e., co-viewers’ likes, gifts, purchases, and new viewers entering the livestreaming room) also inspire positive emotions among viewers [10]. Several studies have shown that mutuality has a significant positive relationship with perceived information usefulness and arousal. For example, high-quality social interaction increases users’ understanding of the platform and products and is less susceptible to interference from sellers or platforms in the context of social commerce. As users receive rich and adequate product information, they perceive the information as useful and have positive emotions, including enjoyment and arousal [70]. Mutuality enables viewers to understand the views of other viewers on the streamer and the product, thereby affecting their cognition and arousing mental models. Therefore, we hypothesize the following:

**Hypothesis** **2a** **(H2a).** *Bullet screen mutuality positively affects consumers’ perceived information usefulness.*

**Hypothesis** **2b** **(H2b).** *Bullet screen mutuality positively affects consumers’ arousal.*

Source credibility is defined as the extent to which an information source is perceived to be believable, competent, and trustworthy by the information recipients [71,72]. The streamer is the viewers’ main source of product information in the context of livestreaming e-commerce. Streamer trustworthiness refers to the degree to which the streamer is regarded to have honesty, integrity, and believability by livestreaming viewers [73]. During the livestreaming of the streamer, the viewers can judge the trustworthiness of the streamer in two ways. First, viewers can judge by the bullet screen information sent by other viewers, likes, gift-giving behaviors, and the performance of other viewers in the process of interacting with the streamer. If the viewers’ evaluation of the streamer is more positive and the behaviors of likes and gifts are more frequent, then the streamer is more credible. Second, viewers can use livestreaming information, such as the number of fans of the streamer and the number of online viewers. A greater number of streamer fans and online viewers is correlated with a higher streamer’s trust. Unlike celebrities or company representatives, government officials who endorse the credibility of the government introduce agricultural products to viewers and send a signal of reliable quality. Government officials pay more attention to the quality of agricultural products to prevent damage to the government’s image. The viewers believe that a higher trustworthiness of the streamer reflects a higher usefulness of the information they perceive. Sussman and Siegal [71] argued that information provided by trusted sources is more useful. Osei-Frimpong et al. [73] believed that the information provided by a trusted spokesperson is more reliable and useful. In addition, honest and trustworthy streamers bring positive emotional experiences to viewers. If the information receivers perceive that the spokesperson is trustworthy, they tend to have more preferences and emotions for the spokesperson and the products endorsed by the spokesperson [74]. Similarly, in the context of livestreaming, when the streamer has a high level of trust, viewers have a positive emotional attitude toward the products they recommend. Therefore, we hypothesize the following:

**Hypothesis** **3a** **(H3a).** *Streamer trustworthiness positively affects consumers’ perceived information usefulness.*

**Hypothesis** **3b** **(H3b).** *Streamer trustworthiness positively affects consumers’ arousal.*

Source expertise refers to the extent to which a source is believed to be capable of making valid assertions [75]. Some consumers may have relatively limited purchasing experience, professional knowledge, and related product information for a certain product; thus, they have a sense of hesitation and uncertainty before purchasing [6]. Viewers can judge the expertise of the streamer through their familiarity with the product and their professional analysis ability of the product. Furthermore, whether the streamer can accurately, objectively, and professionally answer the questions raised by the viewers through the real-time bullet screen can also reflect the professional level of the streamer during the livestreaming. The streamer can clearly convey product-related information through their own high degree of professionalism, including multisource product information, rich practical experience, and a high degree of product knowledge [76,77]. An anchor with professional knowledge can provide a more comprehensive introduction to the product. Source expertise is considered useful, accurate, and reliable because it reduces viewers’ efforts to process product information. For example, suggestions provided by members with higher expertise are more useful in the context of social shopping [75,77]. Biswas, Biswas, and Das [78] found that individuals who are more specialized in high-tech products have more influence on consumer decision making in advertising. In addition, source expertise can promote users to have a positive emotional experience with the recommended products [79,80]. For example, Bhattacherjee and Sanford [81] believed that peripheral route cues (i.e., source expertise) stimulate user’s emotional response. In this study, the streamer vividly introduces the growth environment, freshness, and eating methods of agricultural products to viewers through tasting, and they even show some talents related to the agricultural products (i.e., tea art performances). Complete and professional information on agricultural products not only increases consumers’ knowledge but also improves consumers’ emotional feelings. Therefore, we hypothesize the following:

**Hypothesis** **4a** **(H4a).** *Streamer expertise positively affects consumers’ perceived information usefulness.*

**Hypothesis** **4b** **(H4b).** *Streamer expertise positively affects consumers’ arousal.*

Responsiveness refers to the extent to which consumers receive quick and effective responses from customer service staff or sellers [82,83]. The responsiveness of the streamer is considered a type of direct communication between the streamer and the consumers, eliminating the barriers of time and space in the context of livestreaming e-commerce. The streamer can highly interact with consumers by relying on the real-time and visibility characteristics of the livestreaming platform [8]. During the interaction, consumers can fully understand the product, such as its place of origin, quality, price, and efficacy. At the same time, consumers’ sense of experience also improves. Consumers can get relaxation and pleasure while obtaining product information [11,84]. Katakam et al. [85] believe that the demeanor, language, and skills of the salesperson play an important role in consumers’ handling of product information. Salespersons who use flattery selling tactics and matching incidental similarities are more likely to interact with shoppers. The high level of interaction between salesperson and shoppers enables consumers to obtain complete product information. Hu et al. [86] argued that IPA embedded with machine learning and natural language understanding can analyze and process user behavior and emotional information through a high degree of interaction with users and, in turn, provide a caring, kind, and warm experience. In this research, live streamers can quickly and effectively respond to inquiries or requests from customers in the livestreaming room, including cooking methods and eating methods. Some anchors even humorously respond to the viewers’ questions. The anchor’s real-time response not only improves the convenience and pertinence of communication but also saves the time cost of consumer information acquisition. In addition, frequent interactions prompt consumers to feel disconnected from reality. Therefore, we hypothesize the following:

**Hypothesis** **5a** **(H5a).** *Streamer responsiveness positively affects consumers’ perceived information usefulness.*

**Hypothesis** **5b** **(H5b).** *Streamer responsiveness positively affects consumers’ arousal.*

Perceived usefulness refers to the degree to which a person believes that using a particular system would enhance their job performance [87]. Useful information is more valuable because users can make more informed decisions based on this information. Previous studies have shown that perceived information usefulness is a key variable for predicting users’ purchase intentions. For example, social commerce websites that provide users with high-quality information about products or services they need during transactions increase their shopping intentions by reducing shopping time [88,89]. Racat, Capelli, and Lichy [90] argued that there is a strong association between perceived information usefulness and online purchase intention. Applying this logic, the host can efficiently convey useful information and valuable suggestions to help consumers make purchase decisions, such as product descriptions, purchase procedures, and after-sales services. The more useful viewers perceive the anchor’s information, the more likely they are to buy the products recommended by the anchor. Therefore, we hypothesize the following:

**Hypothesis** **6** **(H6).** *Perceived information usefulness positively affects consumers’ purchase intention.*

Arousal refers to the extent to which people feel excited, alert, stimulated, awakened, and positive in a situation [91]. According to the S–O–R model, the emotional experience generated by the interaction between individuals and the environment will determine their behavioral response. In a pleasant environment, arousal directly leads to the generation of behavioral goals, such as impulse buying [92,93], participation [54], sharing [94]. Previous studies have demonstrated that consumers’ emotional arousal has a positive impact on their purchase intention. Ning Shen and Khalifa [95] found that consumers with positive psychological arousal tend to spend more time and energy to explore the website and purchase products in a pleasant online environment. Chebat and Michon [96] and Barsade et al. [97] believed that well-decorated retail stores can stimulate consumers’ arousal, leading to longer stay in the store and more purchases. In the context of livestreaming e-commerce, consumers who have a good impression of the product or service are more likely to purchase it. When viewers feel that the livestreaming content is comprehensive, accurate, and professional, they tend to have a positive impression of the livestreaming, generating purchase intention. In addition, viewers’ behavior is also susceptible to the influence of other participants. The viewers can judge the attitude of their co-viewers toward the livestreaming based on the comments, gifts, or purchases of other viewers. The positive attitude of co-viewers affects the current audience’s attitude toward livestreaming, thereby stimulating the existing audience to make similar behaviors. Therefore, we hypothesize the following:

**Hypothesis** **7** **(H7).** *Arousal positively affects consumers’ purchase intention.*

Impulsiveness is a personality trait that reflects the difference in the purchasing intentions of consumers to a certain extent [18]. Puri [41] found that consumers with different degrees of impulsiveness can make different behavioral decisions after receiving the same marketing stimulus. Specifically, individuals with a higher level of impulsiveness are more likely to make purchase decisions, while those with lower levels of impulsiveness may lead to fewer purchases. Therefore, individuals with high impulsiveness may not carefully consider or evaluate external stimuli, thus showing unplanned behavior [20]. The moderation of impulsiveness has been confirmed in previous studies [18,34,77]. Chang et al. [18] indicated that the effects of attitude toward purchase intention are positively moderated by impulsiveness in the context of e-commerce. Zafar et al. [77] argued that impulse buying tendency has a moderating effect on consumers’ sentiments and urge to buy impulsively in the context of m-commerce. Wells et al. [34] found that impulsiveness moderates the relationship between information quality and impulse buying. In this study, when highly impulsive consumers perceive that the product information provided in the livestreaming is useful, they are more likely to purchase products without thinking. In addition, highly impulsive individuals are more likely to be awakened by stimuli. Highly impulsive consumers may buy products due to their appeal rather than necessity. Therefore, we hypothesize the following:

**Hypothesis** **8a** **(H8a).** *Impulsiveness positively moderates the relationship between the perceived information usefulness and consumers’ purchase intention.*

**Hypothesis** **8b** **(H8b).** *Impulsiveness positively moderates the relationship between arousal and consumers’ purchase intention.*

**Figure 2:**
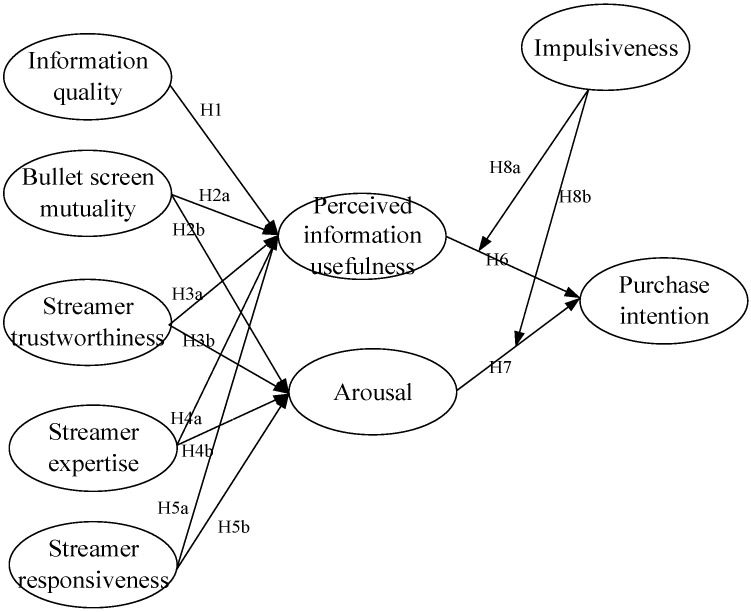
The research model.

Figure 2 shows the proposed research model.

## 4. Method

### 4.1. Measurement Development

All measurement items in this study were adopted from the existing literature. Information quality was adopted from Fu et al. [63]. The measurements of mutuality, streamer responsiveness, and perceived information usefulness were all extracted from Xue et al. [8]. Streamer trustworthiness was measured using the scale from Gao et al. [1]. Streamer expertise was borrowed from Fang [75]. The scale of arousal was obtained from the work of Xu et al. [54]. Purchase intention was adopted from Lu and Chen [6]. Impulsiveness was borrowed from Chopdar et al. [19]. All variables were measured through a seven-point Likert scale (from 1 = strongly disagree to 7 = strongly agree).

Considering that government officials’ livestreaming e-commerce is different from that of celebrities, we modified the measurement items to fit the research context. Two professors and two doctoral students translated the questionnaire items. To ensure that the content of the questionnaire was valid, we invited three government officials who have sold agricultural products through livestreaming and 10 experienced livestreaming shoppers to review the questionnaire. The questionnaire was modified on the basis of the advice of government officials and customers to ensure that the items were clear and accurate.

We used the Credamo platform to conduct pilot tests with 40 live shoppers. The results showed that the Cronbach’s α value (0.83–0.95) and factor loading (above 0.77) of all variables were greater than their threshold values of 0.7 and 0.5, respectively. In addition, the final questionnaire contained six control variables: gender, age, education level, platform type, watching frequency, and following streamers. Table 2 summarizes the measures.

### 4.2. Data Collection

The questionnaire designed in this study was distributed on the Credamo platform. To ensure data quality, we set up a pre-screening question on the platform: “Have you ever watched in the livestreaming of government officials?” Only consumers who had watched the livestreaming room of government officials could continue the questionnaire. The data collection period was from 10 September to 10 October 2021. Users who answered the questionnaire received 5 CNY as a reward. After removing invalid questionnaires, we collected 430 valid questionnaires.

Respondent demographics are presented in Table 3. Of the 430 samples collected, 42.3% were men (*n* = 182) and 57.7% were women (*n* = 248). More than half of the respondents were aged 25–35 (*n* = 262, 60.9%). As for education level, most had a bachelor’s degree (*n* = 331, 77.0%), and most subscribed to streamers’ channels. In terms of platform types, about half of the respondents used e-commerce platforms (*n* = 196, 45.6%), followed by short video platforms (*n* = 189, 44.0%). In terms of viewing frequency, more than half of the respondents watched livestreaming one to six times a week (*n* = 192; 52.5%).

Harman’s one-factor test was used to evaluate the common method bias (CMB). The results show that the explanatory power of the first factor was 28.63% (less than 50%), indicating that no single factor could explain most of the variance. We also used a variance inflation factor (VIF) to assess collinearity issues. The results showed that the VIF ranged from 1.773 to 2.536 (below 3). This indicates that there was no obvious collinearity problem in this study.

## 5. Result

### 5.1. Measurement Model

This study used AMOS 24.0 to analyze the data. We first measured the measurement model, including reliability, convergent validity, and discriminant validity. Table 4 and Table 5 show the results of the measurement model. The results show that the Cronbach’s alphas of all variables were greater than 0.7 and the composite reliability (CR) value was greater than 0.8, indicating that the measurements had high reliability [98]. The average extracted variance (AVE) value of all constructs was greater than 0.5, indicating that the measurements had good convergent validity [98]. Discriminant validity was also met as no intercorrelation of the constructs exceeded the square root of the AVE of either of the compared constructs [99]. Table 6 shows that the values of all constructs were lower than 0.85, indicating that this study had good discriminant validity [100].

### 5.2. Structural Models

**Figure 3:**
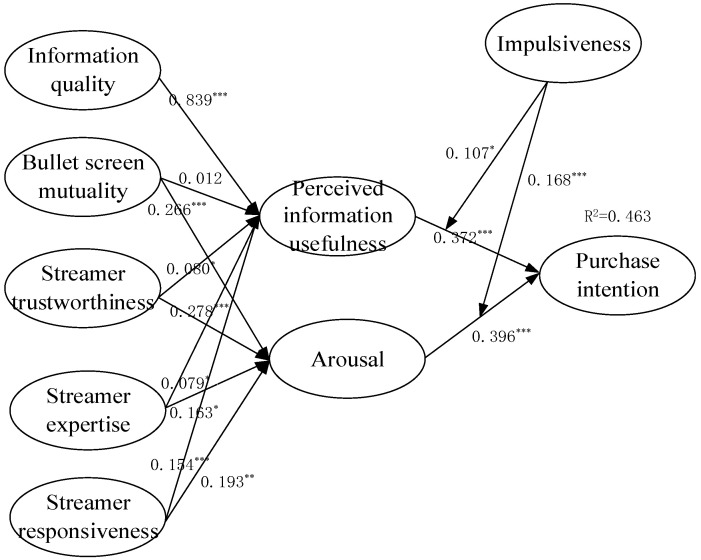
Model testing results. Note: *** *p* < 0.001, ** *p* < 0.01, * *p* < 0.05.

Before testing the hypothetical path, we first calculated the fitting index of the structural model. The results were as follows: chi-square (χ^2^) = 750.172, degrees of freedom (df = 384), χ^2^/df = 1.954, *p*-value = 0.000, NFI = 0.934, RFI = 0.925, IFI = 0.967, TLI = 0.962, CFI = 0.967, RMSEA = 0.047. Considering that the value of χ^2^/df was lower than 3.0, the NFI, RFI, CFI, IFI, and TLI were above 0.90, and the value of RMSEA was less than 0.08, the research model and the sample data had a good fit. Table 7 and Figure 3 illustrate the standardized path coefficients. Except for H2a, all the hypotheses were significant. H1 was supported, indicating that information quality (β = 0.839, *p* < 0.001) was positively associated with perceived information usefulness. Regarding the streamer dimension, the streamer responsiveness (β = 0.154; *p* < 0.001) was the most influential variable, followed by streamer trustworthiness (β = 0.080; *p* < 0.05) and streamer expertise (β = 0.079; *p* < 0.05). On the other hand, the effect of bullet screen mutuality on perceived information usefulness was not statistically significant. Bullet screen mutuality, streamer trustworthiness, streamer expertise, and streamer responsiveness were positively associated with arousal. The influence of perceived information usefulness (β = 0.372; *p* < 0.001) and arousal (β = 0.396; *p* < 0.001) on purchase intention was supported.

**Figure 4:**
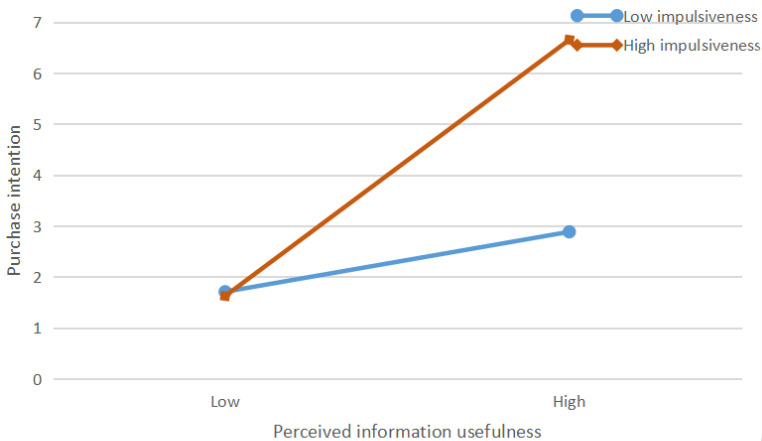
The interaction effect of perceived information usefulness and impulsiveness on purchase intention.

**Figure 5:**
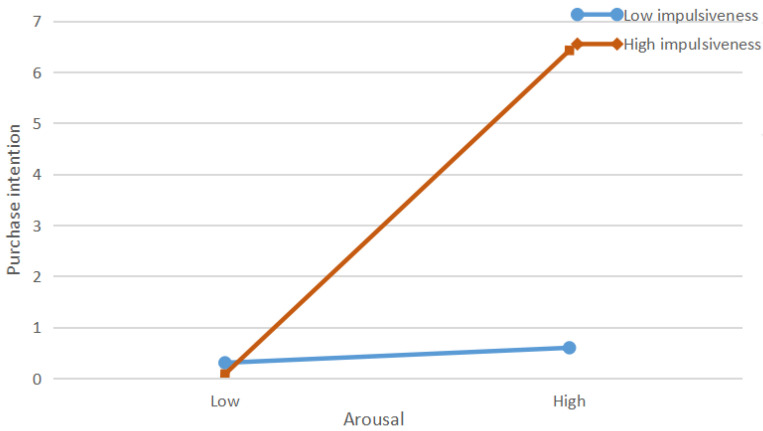
The interaction effect of arousal and impulsiveness on purchase intention.

Table 8 shows the results of the moderating effects of impulsiveness. The results indicated that impulsiveness significantly and positively moderated the relationship between perceived information usefulness (β = 0.107; *p* < 0.05) and purchase intention. Thus, H8a was supported. Impulsiveness, on the other hand, significantly and positively moderated the relationship between arousal (β = 0.168; *p* < 0.001) and purchase intention. Thus, H8b was supported. We conducted a simple slope analysis to further understand the moderating effect (see Figure 4 and Figure 5). Figure 4 depicts the interaction of perceived information usefulness and impulsiveness. Perceived information usefulness had a significant positive effect on purchase intention under high- and low-impulsiveness conditions. This suggested that impulsiveness positively moderated the relationship between perceived information usefulness and purchase intention. Figure 5 depicts the interaction of arousal and impulsiveness. Similarly, Figure 5 shows that arousal had a stronger effect on purchase intention when impulsiveness was high.

## 6. Discussion

### 6.1. Research Findings

Drawing upon the S–O–R framework, this study explored whether government officials’ livestreaming selling increases consumers’ purchase intentions. In this study, platform factor (information quality), user factor (bullet screen mutuality), and streamer factors (streamer trustworthiness, streamer expertise, and streamer responsiveness) were identified as the antecedents that affect viewers’ information processing and emotions in livestreaming e-commerce. Moreover, this study examined the effects of perceived information usefulness and arousal on purchase intention. In addition, the moderation effect of impulsiveness was examined. Altogether, 12 of the 13 hypotheses were supported. We found some interesting findings.

Information quality exerted significant effects on perceived information usefulness (H1). The result showed that the streamer provided timely, comprehensive, and detailed product information to the viewers through the form of video. Viewers who obtain high-quality product information can effectively evaluate products and make informed decisions. This finding is consistent with Meilatinova [60], Fu et al. [63], and Kang et al. [11], which indicates that high-quality information can reduce consumers’ time and effort when shopping. Contrary to our expectations, bullet screen mutuality had nonsignificant effects on perceived information usefulness (H2a). Two reasons may explain this result. First, the bullet screen comments that scroll in real-time on the screen make the earlier comments quickly replaced by the later comments. Viewers who cannot see the answer will quickly turn to ask the streamer. Second, comments presented in text are less attractive than streamer responses presented in video. Viewers are more inclined to consult the streamer directly and acquire personalized and replies. Bullet screen mutuality exerted significant effects on arousal (H2b). Real-time scrolling bullet screen comments, continuous pop-up gifts, and shopping tips set off a lively livestreaming atmosphere, which in turn stimulates the viewer to have a positive emotional response to the livestreaming room.

Our findings confirmed that streamer enablers, namely, streamer trustworthiness (H3), streamer expertise (H4), and streamer responsiveness (H5), had significant positive effects on consumers’ perceived information usefulness and arousal. These findings are compatible with previous studies [73,79,85]. Government officials who endorse credibility pay more attention to product quality to prevent damage to the government’s image. Unlike the serious image in daily government work, the trained government officials convey product-related information to the viewer in an image that is close to the people. The streamer promotes the flow of information and emotions through professional analysis and timely response to agricultural products, including growth environment, taste, and eating methods. Therefore, streamer trustworthiness, streamer expertise, and streamer responsiveness are important antecedents of perceived information usefulness and arousal in livestreaming e-commerce.

The empirical results confirmed that perceived information usefulness (H6) and arousal (H7) had a significant positive effect on purchase intention. These results are compatible with previous studies [88,95]. Useful information reduces the uncertainty of consumers’ perception of product quality. The more useful the information is provided by the streamer, the more likely consumers are to buy the products recommended by the streamer. In the livestreaming environment, the real-time scrolling bullet screen and the likes, gift-giving, and purchase behavior of the viewers in the livestreaming room set off a warm livestreaming atmosphere, thereby stimulating the new viewers in the livestreaming room to perform a similar behavior, i.e., purchase intention. In addition, the research findings further confirmed that impulsiveness had a positive and significant moderating effect between perceived information usefulness and purchase intention and between arousal and purchase intention. These findings indicate that when highly impulsive consumers perceive that the streamer provides high-quality useful information, they are more likely to produce purchase intentions. Highly impulsive individuals are also more likely to be aroused by stimuli and purchase products without thinking.

### 6.2. Theoretical Implications

This research made significant theoretical contributions to the research fields of information system, livestreaming, and impulsiveness by exploring the relationship between government officials’ livestreaming e-commerce and consumers’ purchase intention. First, this is one of the early studies on the application of the S–O–R model to the field of livestreaming e-commerce. Although some theories (i.e., signaling theory, ELM, U&G, and affordance theory) have been used to study consumers’ purchase intention, there have been few studies using the S–O–R framework to date [1,6,13,22,25]. How livestreaming e-commerce, which is different from other backgrounds, promotes consumers’ purchase intentions is not yet known. This study enriches the S–O–R and livestreaming e-commerce literature.

Second, this study examined the effect of the characteristics of the streamer on consumers’ purchase intention from the perspective of government officials as streamers. Existing research on livestreaming e-commerce mainly explored the antecedents of consumers’ purchase intention from the perspective of Internet celebrity anchors [9,11,12]. However, there are essential differences between government officials and Internet celebrities in livestreaming selling. The purpose of government officials’ livestreaming e-commerce is to increase farmers’ income and help them avoid poverty, while that of Internet celebrities is to make money. In addition, this study explored consumer purchase intentions from the perspective of agricultural and sideline products. Previous studies rarely considered specific product categories [6]. Therefore, this study supplemented the livestreaming e-commerce literature from the perspective of government officials and agricultural and sideline products.

Third, we also examined the moderating effect of impulsiveness. As a typical personal trait, there are objective differences in the impulsive traits of different individuals. Although impulsiveness has been studied in various contexts (i.e., e-commerce, mobile commerce, and social commerce) [18,19], little is known about how impulsiveness moderates the effect of perceived information usefulness and arousal on purchase intention in the context of livestreaming e-commerce. Our research results indicated that a higher impulsiveness of individuals was related to a stronger their intention to buy products. So far, this study is one of the earlier studies to investigate the moderating effect of impulsiveness on consumers’ purchase intention. Therefore, this study expands the scope of the impulsiveness literature.

### 6.3. Practical Implications

This study provides some practical suggestions for government officials who livestreaming selling and marketers. Firstly, to improve the viewer’s perceived information usefulness, the streamer should pay attention to the information quality. Consumers rely more on the product information provided by the streamer to make decisions because live shopping has the characteristics of virtuality, immediacy, and sociality. The more comprehensive, complete, and detailed the product information is provided by the streamer, the less uncertainty and doubts the viewer will have. Therefore, the streamer should exert substantial efforts to display and convey complete information, and then guide the viewer to make wise decisions.

Secondly, streamer trustworthiness is the key to consumers’ purchase intention. The official identity of the streamer can be used as a publicity point for the livestreaming selling by government officials. At the same time, it should also take the credit endorsement as the bottom line. This means that government officials should make product selection, pricing, logistics, and after-sales planning before livestreaming. Government officials should strictly select products to avoid damage to the government’s image.

Thirdly, the professional ability of government officials livestreaming selling needs to be strengthened. Government officials livestreaming e-commerce should be familiar with product-related professional knowledge and provide consumers with more accurate product information. Consumers can make informed decisions through a deeper understanding of products. In addition, the streamer can provide the viewer with coupons, discounts, and lotteries during the livestreaming e-commerce. The warm livestreaming atmosphere can also increase consumers’ purchase intention.

Lastly, the packaging of agricultural and sideline products can be made of new materials of recycling and reuse, which can enhance ecological sustainability and increase environmental awareness of consumers. Designers who design packaging and select materials need to be trained in eco-design to understand the ecological illusion, so that they can correctly apply methods and avoid repeated mistakes [101]. When government officials are broadcasting, they can show the information of green products and packaging and how green products and packaging contribute to health and the environment. In addition, governments can establish the credibility of eco-label information for sustainable products. They can enhance the effectiveness of sustainable products by emphasizing the environmental benefits of green products, encouraging sustainable lifestyles, enhancing the image of green brands, and minimizing the inherent flaws of green products.

### 6.4. Limitations and Future Research

Our study had several limitations. First, the product types were restricted to agricultural and sideline products. With the explosive growth of e-commerce livestreaming, various products continue to enter the livestreaming platform, such as clothing, cosmetics, and digital products. Future research should apply the model to other scenarios to validate whether it has good external validity. Second, this study did not consider the possible differences between different types of livestreaming commerce platforms. Each platform has unique characteristics. For example, Vipshop.com mainly sells clothing. Future research should explore the differential impact of livestreaming platform types on users’ purchase intentions. Third, this study explored gender and age as control variables. Previous research proposed that gender and age affect consumers’ purchase intentions. In addition, the gender and age of the streamer also affect consumers’ purchase intentions. Future research should explore the purchasing intentions of consumers of different ages and genders in the context of streamers of different ages and genders. Fourth, this study used cross-sectional data, but users’ purchase intentions evolve dynamically over time. Future research should use data mining technology to crawl and analyze the bullet screen and audience behavior of government officials’ livestreaming e-commerce, and then reveal the effect of streamer language style, product price, and platform type on sales performance. Fifth, this study was conducted in China. However, consumers’ intentions and behaviors may vary from culture to culture. Future research can focus on cross-cultural comparative research in the context of livestreaming e-commerce, which would benefit the development of international marketplaces. Sixth, future research could investigate other independent variables (i.e., streamer attractiveness, co-viewer involvement and background visual complexity) and moderating variables (i.e., mindfulness, brand familiarity) to expand the research model.

## 7. Conclusions

The Chinese government has taken a series of measures to develop the rural economy and increase farmers’ income. Livestreaming e-commerce, as an innovative shopping channel, is experiencing unprecedented growth. To help agricultural products sell well, government officials endorsed by government credibility have begun to sell goods through live broadcasting. As a new measure to help farmers, its application effect is still unknown. Therefore, this study explored the influence of platform, user, and streamer factors on consumers’ purchase intentions by applying the S–O–R framework. It also investigated the moderating role of impulsiveness. The findings showed that information quality, streamer trustworthiness, streamer expertise, and streamer responsiveness positively affect purchase intention through perceived information usefulness and arousal. This showed that consumers value information quality and anchor characteristics during shopping. Impulsiveness positively moderates the relationship between perceived information usefulness and purchase intention and between arousal and purchase intention. It can be inferred that consumers with high impulsivity traits have stronger purchase intentions. This study offers the view that government officials’ livestreaming increases consumers’ purchase intention, enriches the livestreaming e-commerce literature, and promotes the understanding of the S–O–R framework in the marketing field.

## Figures and Tables

**Table 1:** The difference between livestreaming e-commerce and e-commerce.

Attributes	Livestreaming E-Commerce	E-Commerce
Supporting platform	E-commerce website, livestreaming platform, brand official website	E-commerce website
Interactive tools	Bullet screen real-time interaction	Chat software message
Interactive form	One person to one person, one person to many	One person to one person
Interactive object	Seller–platform–consumer	Seller-consumer
Information form	Picture, text, video	Picture, text
Information sources	Not editable	Editable
Experience mode	Online experience	No experience
Purchase method	Buy while watching	Final purchase
Payment method	Online payment	Online payment
Service time	Unlimited	Unlimited
Marketing content	Personalized	Fixed

**Table 2:** Measures of constructs.

Variable	Items	Source
Information quality (IQ)	IQ1. Information in the livestreaming of government officials was accurate on the item that I wanted to purchase.	Fu et al. [63]
	IQ2. Overall, I think the livestreaming of government officials provided useful information.	
	IQ3. Information in the livestreaming of government officials was reliable.	
	IQ4. Information in the livestreaming of government officials was up-to-date.	
	IQ5. The livestreaming of government officials provided sufficient information when I tried to make a transaction.	
Mutuality (MU)	MU1. When watching live broadcasting of government officials, I can get a lot of advice and help from other buyers’ comments showed by anchors.	Xue et al. [8]
	MU2. When watching live broadcasts of government officials, I can offer my shopping knowledge and experience by sending barrages and interacting with anchors and other buyers.	
	MU3. When watching live broadcasts of government officials, I can read the barrages sent by anchors and other buyers to help myself make purchase decisions.	
Streamer trustworthiness (ST)	ST1. Government officials as the streamers know the products that they recommend very well.	Gao et al. [1]
	ST2. Government officials as the streamers know how to use products they recommend very well.	
	ST3. I think government officials as streamers are honest.	
	ST4. I think government officials as streamers are reliable.	
Streamer expertise (SE)	SE1. Government officials as streamers are very knowledgeable in evaluating quality of product.	Fang [75]
	SE2. Government officials as streamers are experts in evaluating quality of product.	
	SE3. Government officials as streamers are highly experienced in consuming the product.	
Streamer responsiveness (SR)	SR1. Government officials as streamers are very happy to communicate with me.	Xue et al. [8]
	SR2. Government officials as streamers can answer my questions and requests in time.	
	SR3. The response of government officials as streamers is closely related to my problems and requests.	
	SR4. Government officials as streamers can provide relevant information for my inquiry in time.	
Perceived usefulness (PU)	PU1. Participating in live interactions, I can get useful knowledge and information on commodities.	Xue et al. [8]
	PU2. Participating in live interactions, I can obtain an exciting and useful shopping information on commodities.	
	PU3. Participating in live interactions can save my time and improve shopping efficiency.	
Arousal (AR)	AR1. I feel enthusiastic about taking action while watching the livestreaming of government officials (e.g., shopping or social sharing).	Xu et al. [54]
	AR2. I feel exhilarated to participate during the livestreaming of government officials.	
	AR3. I feel energized to initiate a variety of behaviors (suggestions/responses) during the livestreaming of government officials.	
	AR4. I feel excited about engaging with the livestreaming of government officials.	
Purchase intention (PI)	PI1. I am very likely to buy the products from the livestreaming of government officials.	Lu and Chen [6]
	PI2. I would consider buying the products from the livestreaming of government officials in the future.	
	PI3. I intend to buy the products from the livestreaming of government officials.	
Impulsiveness (IM)	IM1. When I shop in the livestreaming of government officials room, I often buy things spontaneously.	Chopdar et al. [19]
	IM2. When I shop in the livestreaming of government officials room, I often buy things without thinking beforehand.	
	IM3. When I shop in the livestreaming of government officials room, sometimes I feel like buying things on the spur of the moment.	
	IM4. “Just do it” describes the way I buy things in the livestreaming room of government officials.	

**Table 3:** Details of respondents.

Category		Frequency	Percentage
Gender	Male	182	42.3%
	Female	248	57.7%
Age	Under 18	18	4.2%
	19–24	47	10.9%
	25–35	262	60.9%
	36–45	57	13.3%
	46–55	26	6.0%
	55 older	20	4.7%
Education level	High school or below	19	4.4%
	Junior college	50	11.6%
	Bachelor	331	77.0%
	Postgraduate or above	30	7.0%
Are you following streamers?	Yes	347	80.7%
	No	83	19.3%
Platform types	E-commerce platform(e.g., TaoBao, JingDong)	196	45.6%
	Social Media platform(e.g., Sina Microblog)	45	10.5%
	Short video platform(e.g., Tiktok, KuaiShou)	189	44.0%
Watch frequency	Every day	112	30.6%
	1–6 times a week	192	52.5%
	1–3 times a month	47	12.8%
	Less than once a month	15	4.1%

**Table 4:** Convergent validity and reliability analysis.

Constructs	Items	Factor Loading	CR	AVE	Cronbach’s Alpha
Information quality	IQ1	0.904	0.961	0.831	0.961
	IQ2	0.928			
	IQ3	0.910			
	IQ4	0.907			
	IQ5	0.910			
Mutuality	MU1	0.806	0.840	0.642	0.800
	MU2	0.614			
	MU3	0.949			
Streamer trustworthiness	ST1	0.755	0.843	0.578	0.838
	ST2	0.568			
	ST3	0.843			
	ST4	0.843			
Streamer expertise	SE1	0.791	0.911	0.720	0.910
	SE2	0.867			
	SE3	0.905			
	SE4	0.826			
Streamer responsiveness	SR1	0.790	0.906	0.707	0.905
	SR2	0.887			
	SR3	0.848			
	SR4	0.836			
Perceived usefulness	PU1	0.904	0.933	0.823	0.933
	PU2	0.907			
	PU3	0.911			
Arousal	AR1	0.787	0.861	0.607	0.860
	AR2	0.799			
	AR3	0.777			
	AR4	0.753			
Purchase intention	PI1	0.820	0.888	0.725	0.887
	PI2	0.853			
	PI3	0.881			
Impulsiveness	IM1	0.905	0.948	0.821	0.948
	IM2	0.912			
	IM3	0.922			
	IM4	0.885			

IQ, information quality; MU, mutuality; ST, streamer trustworthiness; SE, streamer expertise; SR, streamer responsiveness; PU, perceived information usefulness; AR, arousal; PI, purchase intention; IM, impulsiveness.

**Table 5:** Discriminant validity analysis.

Construct	IQ	MU	ST	SE	SR	PU	AR	PI	IM
IQ	0.912								
MU	0.638	0.801							
ST	0.456	0.348	0.760						
SE	0.555	0.500	0.606	0.849					
SR	0.620	0.485	0.500	0.579	0.841				
PU	0.676	0.610	0.433	0.558	0.653	0.907			
AR	0.536	0.474	0.508	0.521	0.515	0.555	0.779		
PI	0.546	0.519	0.510	0.635	0.536	0.533	0.503	0.851	
IM	0.626	0.418	0.301	0.417	0.436	0.611	0.395	0.451	0.906

IQ, information quality; MU, mutuality; ST, streamer trustworthiness; SE, streamer expertise; SR, streamer responsiveness; PU, perceived information usefulness; AR, arousal; PI, purchase intention; IM, impulsiveness.

**Table 6:** Heterotrait–monotrait ratios (HTMT).

.	IQ	MU	ST	SE	SR	PU	AR	PI	IM
IQ	-								
MU	0.733	-							
ST	0.507	0.420	-						
SE	0.589	0.577	0.696	-					
SR	0.662	0.565	0.572	0.632	-				
PU	0.747	0.713	0.474	0.602	0.709	-			
AR	0.586	0.564	0.596	0.586	0.581	0.622	-		
PI	0.591	0.615	0.593	0.821	0.599	0.582	0.577	-	
IM	0.653	0.472	0.336	0.444	0.466	0.643	0.434	0.489	-

IQ, information quality; MU, mutuality; ST, streamer trustworthiness; SE, streamer expertise; SR, streamer responsiveness; PU, perceived information usefulness; AR, arousal; PI, purchase intention; IM, impulsiveness.

**Table 7:** SEM standardized regression path analysis.

Hypothesis	Standardized Estimate β	*p*-Value	Hypothesis Testing Result
H1: information quality → perceived information usefulness	0.839	0.000 ***	Supported
H2a: bullet screen mutuality → perceived information usefulness	0.012	0.755	Not supported
H2b: bullet screen mutuality → arousal	0.266	0.000 ***	Supported
H3a: streamer trustworthiness → perceived information usefulness	0.080	0.014 *	Supported
H3b: streamer trustworthiness → arousal	0.278	0.000 ***	Supported
H4a: streamer expertise → perceived information usefulness	0.079	0.028 *	Supported
H4b: streamer expertise → arousal	0.163	0.019 *	Supported
H5a: streamer responsiveness → perceived information usefulness	0.154	0.000 ***	Supported
H5b: streamer responsiveness → arousal	0.193	0.002 **	Supported
H6: perceived information usefulness → purchase intention	0.372	0.000 ***	Supported
H7: arousal → purchase intention	0.396	0.000 ***	Supported

Note: *** *p* < 0.001, ** *p* < 0.01 * *p* < 0.05.

**Table 8:** Moderation test results.

	**Main Effect**	**Interaction Effect**
Perceived information usefulness	0.410 ***	0.349 ***
Impulsiveness	0.200 ***	0.187 ***
Perceived information usefulness × impulsiveness		0.107 *
R^2^	0.309	0.305
ΔR^2^		0.310
*p*-Value		0.044
	**Main Effect**	**Interaction Effect**
Arousal	0.385 ***	0.293 ***
Impulsiveness	0.299 ***	0.307 ***
Arousal × impulsiveness		0.168 ***
R^2^	0.329	0.326
ΔR^2^		0.344
*p*-Value		0.000

Note: *** *p* < 0.001, * *p* < 0.05.

## Data Availability

The data that support the findings of this study are available from the corresponding author, upon reasonable request.
